# Human Polyomavirus Related to African Green Monkey Lymphotropic Polyomavirus

**DOI:** 10.3201/eid1708.110278

**Published:** 2011-08

**Authors:** Virginie Sauvage, Vincent Foulongne, Justine Cheval, Meriadeg Ar Gouilh, Kevin Pariente, Olivier Dereure, Jean Claude Manuguerra, Jennifer Richardson, Marc Lecuit, Ana Burguière, Valérie Caro, Marc Eloit

**Affiliations:** Author affiliations: Institut Pasteur, Paris, France (V. Sauvage, M. Ar Gouilh, K. Pariente, J.C. Manuguerra, M. Lecuit, A. Burguière, V. Caro, M. Eloit);; Institut National de la Santé et de la Recherche Médicale, Montpellier, France (V. Foulongne, O. Dereure);; University of Montpellier, Montpellier (V. Foulongne, O. Dereure);; Pathoquest, Paris (J. Cheval, M. Eloit);; Ecole Nationale Vétérinaire d’Alfort, Maisons Alfort, France (J. Richardson, M. Eloit);; Paris Descartes University, Paris (M. Lecuit)

**Keywords:** polyomavirus, viruses, skin, infections, shedding, African green monkey lymphotrophic virus, Merkel cell carcinoma patients, high-throughput nucleotide sequencing, expedited, research

## Abstract

While studying the virome of the skin surface of a patient with a Merkel cell carcinoma (MCC) by using unbiased, high-throughput sequencing, we identified a human polyomavirus nearly identical to human polyomavirus 9, a virus recently reported in blood and urine of renal transplantion patients and closely related to the African green monkey lymphotropic polyomavirus. Specific PCR analysis further identified this virus in 2/8 patients with MCC but in only 1/111 controls without MCC. This virus was shed for >20 months by the MCC index patient and was on the skin of the spouse of the index patient. These results provide information on the viral ecology of human skin and raise new questions regarding the pathology of virus-associated skin disorders.

## Abstract 2

TOC summary: This virus is shed at the human skin surface.

*Polyomaviridae* is a family of nonenveloped viruses with a circular double-stranded DNA genome. Natural hosts for *Polyomaviridae* are primates, including humans and monkeys, cattle, rabbits, rodents, and birds (*1*). Currently, viruses in this family that infect humans are the opportunistic JC polyomavirus (JCPyV) associated with progressive multifocal leukoencephalopathy in immunocompromised person; BK polyomavirus (BKPyV) associated with interstitial nephritis and hemorrhagic cystitis; KI polyomavirus (KIPyV) identified in respiratory secretions of patients with respiratory symptoms at the Karolinska Institute (Stockholm, Sweden); WU polyomavirus (WUPyV), isolated from patients with similar symptoms at Washington University (St. Louis, MO, USA); Merkel cell polyomavirus (MCPyV), associated with the rare but aggressive Merkel cell carcinoma (MCC); and trichodysplasia spinulosa–associated polyomavirus (TSPyV), associated with a rare skin condition in immunocompromised persons (*2*).

Two recently identified polyomaviruses, human polyomaviruses 6 and 7 (HPyV6 and HPyV7) have been detected in cutaneous swab specimens of healthy persons (*3*). HPyV9 has been identified by consensus PCR in blood and urine of asymptomatic renal transplant recipients (*4*). In this study of the skin virome of a patient with MCC, using high-throughput sequencing (HTS) and comparing sequences from a patient with MCC with sequences from healthy controls, we identified a human polyomavirus strain nearly identical to HPyV9, a virus species closely related to the lymphotropic polyomavirus (LPV).

## Methods

### Patients and Sample Collection

For analysis by HTS, 6 DNA samples extracted from cutaneous swabs obtained from the skin surface of facial areas (forehead and eyebrows) of patients previously studied by PCR for MCPyV sequences were selected (*5*). These samples included 1 from an index patient with an MCC on his elbow and 5 from the skin of 5 healthy persons.

For investigation of prevalence by specific nested PCR, 120 skin specimens were similarly obtained from 120 volunteers. The median age of these persons was 48 years (range 19–96 years); 30 persons were 57–96 years of age (median age 71 years). This group of 120 volunteers was composed of 40 patients hospitalized or attending outpatient clinics at the dermatology unit at Montpellier University Hospital for various skin disorders (including 8 patients with MCC; median age 75 years, range 57–86 years), 20 immunocompromised patients without skin lesions (10 patients infected with HIV-1 without skin symptoms and 10 renal transplant recipients receiving immunosuppressive regimens [steroids, mycophenolate mofetil, and calcineurin inhibitors]), and 60 healthy controls.

Respiratory samples tested were composed of 46 bronchoalveolar lavage samples obtained from hospitalized patients in intensive care units with acute respiratory failure of unknown origin and 46 nasopharyngeal aspirates from children in the pediatric emergency unit at Montpellier University Hospital with various respiratory tract disorders. An additional 92 fecal samples were obtained from children hospitalized in the pediatric unit for gastroenteritis.

### Extraction and Amplification of DNA

DNA from all samples was extracted as described (*5*). For HTS, DNA was amplified by using a bacteriophage ϕ29 polymerase-based rolling circle amplification assay and random primers. The protocol of the QIAGEN REPLI-g Midi Kit (QIAGEN, Courtaboeuf, France) was followed as recommended by the manufacturer.

### High-Throughput Sequencing

HTS was performed by using the Illumina HiSeq 2000 apparatus (Illumina Inc., San Diego, CA, USA) at GATC Biotech AG (Konstanz, Germany). Five micrograms of high molecular weight amplified DNA was divided into 200–350-nt fragments to which adaptors were ligated. These adaptors included a nucleotide tag that enabled multiplexing several samples per lane or channel. Sequencing was conducted at a mean depth per sample of 8.9 × 10^6^ paired-end reads of 100 nt (range 7.6–10.3 × 10^6^ reads).

### Sequence Analysis

Sequences were first sorted by using a subtractive database comparison procedure. Several assembly programs dedicated to short or medium reads were used to generate contigs: Velvet (www.ebi.ac.uk/~zerbino/velvet), SOAPdenovo (http://soap.genomics.org.cn), and CLC Genomics Workbench (www.clcbio.com) (J. Cheval et al., unpub. data). Comparison of single reads and contigs with known genomic and taxonomic data was performed by using dedicated specialized viral, bacterial, and generalist databases created and maintained at the Institut Pasteur (GenBank viral and bacterial databases). Aforementioned databases were screened by using BLASTN and BLASTX (http://blast.ncbi.nlm.nih.gov/Blast.cgi). We used BLAST software (Paracel, Pasadena, CA, USA) capable of executing searches on multiple nonshared memory processors simultaneously.

The entire sequence of the Institut Pasteur polyomavirus (IPPyV) strain genome was analyzed and annotated by using CLC Genomics Workbench (CLC Bio, Aarhus, Denmark). GenBank reference sequences of other members of the family *Polyomaviridae* used were JCPyV (NC_001699), BKPyV (NC_001538), KIPyV (NC_009238), WuPyV (NC_009539), MCPyV (NC_010277), SV40 (NC_001669), TSPyV (NC_014361), and LPV (M30540). Protein structures were visualized by using Pymol (Delano Scientific LLC, San Francisco, CA, USA).

### Phylogenetic Analysis

Phylogenetic reconstructions were based on separate analyses of nucleotide sequences from viral protein 1 (VP1) and large T antigen (LT). A 974-nt region of monkey B-lymphotropic papovavirus (reference sequence M30540.1 from the VP1 coding sequence) was aligned with corresponding regions from the polyomaviruses available in GenBank. For the LT matrix, a 1,453-nt region (same reference sequence as for VP1) was used for analysis. Sequences were aligned by using SeaView version 4 (*6*) and the Muscle algorithm (*7*). Only partial but contiguous parts of each alignment were included in final matrixes because alignment in some regions was not possible. The 742–1715 and 2904–4356 nt regions were included in matrixes. The matrixes were based on monkey B-lymphotropic papovavirus M30540 and African green monkey polyomavirus NC_004763 sequence annotations for VP1 and LT genes, respectively.

Phylogenetic analyses were performed by using a probabilistic (Bayesian) approach implemented in BEAST (*8*). Matrixes were tested against 88 substitution models by using jModelTest software (*9*). On the basis of results obtained with jModelTest software, the generalized time reversible substitution model (with invariant sites and a gamma site heterogeneity distribution) was used for analysis. The 3-codon partition model of evolution and the Yule speciation process were also specified as priors. A Markov chain Monte Carlo method was used to set 30,000,000 states to obtain an adequate posterior effective sample size >300. Pertinence of nodes was evaluated by using posterior probabilities. Sequences used in phylogenetic reconstructions were obtained from the National Center for Biotechnology Information (NCBI) database (www.ncbi.nlm.nih.gov/nucleotide). Confidence intervals for proportions were calculated according to the efficient-score method (corrected for continuity) (*10*) (http://dogsbody.psych.mun.ca/VassarStats).

### PCR

For sequencing the IPPyV by the Sanger method, 9 primer pairs were designed to amplify the entire genome by reference to the contigs assembled from HTS data acquired in the first phase (see Results). Primer sequences and protocols are available upon request. After the genome was sequenced, we developed a specific nested PCR for detection of IPPyV in samples by using primers based on the IPPyV genome sequence and designed by using PrimerPro 3.4 software (www.changbioscience.com): VP1_354F (5′-ACCATATCAGTAGGATAGGTA-3′) and VP1_354R (5′-TGAATTGTATGGCTACAGTGC-3′) for the outer PCR, and VP1_198F (5′-CACTGGGATAGTTCCTGAGG-3′) and VP1_198R (5′-CCTAATGCTACTACCCTCCCT-3′) for the inner PCR. These primers were designed to avoid amplification of other known human polyomaviruses.

### Ethical Approval

The study was reviewed and approved by the Institut Pasteur Comité de Recherche Clinique and the French Commission Nationale Informatique et Libertés (09.465). Consent was provided by participants for obtaining human samples according to French regulations.

## Results

### Identification of the IPPyV Strain of HPyV9

Using 8,052,770 Illumina reads obtained from DNA extracted from the skin surface of the MCC index patient, we assembled the complete genome of MCPyV. We found numerous papillomavirus contigs and contigs covering more than half of the genomes of HPyV6 and HPyV7. Additionally, 14 other contigs were assembled that showed a better homology with LPV (NCBI accession no. M30540, version M30540.1, GI:333282) than with any other virus present at that time in the NCBI database, including other human or animal members of the family *Polyomaviridae*. On the basis of sequence of 8 of the 14 obtained contigs, which were distributed along the LPV genome, we defined a set of 9 primer pairs encompassing the entire target genome. These primers enabled amplification of the entire genome by PCR and analysis of its sequence of 5,028 nt by using the Sanger method, which confirmed the circular nature of the genome.

**Figure 1:**
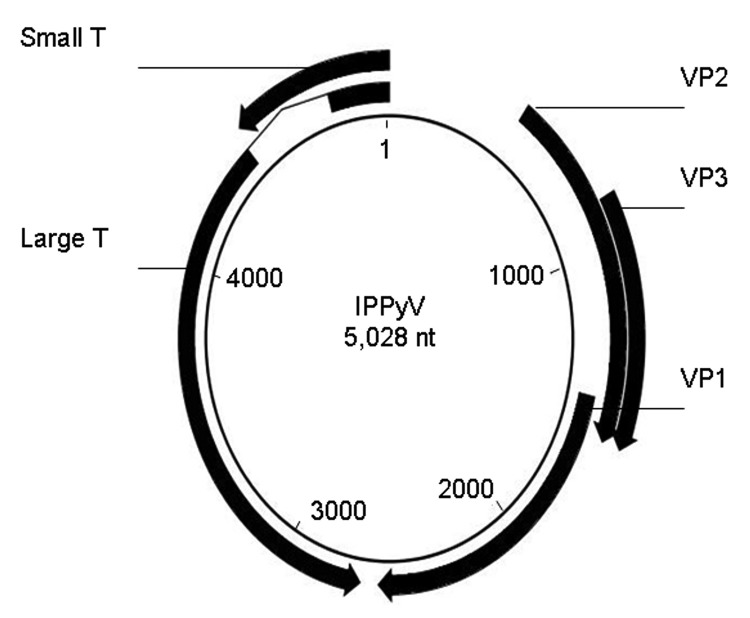
Genomic map of the circular genome of the Institut Pasteur polyomavirus (IPPyV) strain of human polyomavirus 9. Arrows indicate open reading frames. Small T, small T antigen; VP, viral protein; Large T, large T antigen.

Whole genome organization of the IPPyV strain, which exhibits general molecular characteristics of polyomaviruses, is shown in Figure 1. It encodes analogs of small T antigen, LT, and structural proteins VP1, VP2, and VP3 and does not appear to encode an agnoprotein. Pairwise amino acid identity was 100% between IPPyV and HPyV9 proteins, 72%–80% between IPPyV and LPV proteins, and much lower for other known family *Polyomaviridae* members (Table). Because the nucleotide sequence of IPPyV is nearly identical to that of HPyV9, with a difference of only 2 nt in a noncoding region at nt 4449, it appears that IPPyV should be considered a strain of HPyV9. Its sequence have been was deposited in GenBank (accession no. FR823284).

**Table:** Amino acid identity between putative proteins encoded by IPPyV and proteins of *Polyomaviridae* deduced by using pairwise sequence alignment*

Protein	Putative open reading frame	Frame	No. amino acids	Amino acid identity, %								
				JCV	BKV	KIV	WuV	MCyV	TSV	SV40	LPV	HPyV9
VP1	1443–2558	+3	371	53.9	53.2	28.3	28.3	54.8	60.6	52.,9	87.1	100.0
VP2	503–1561	+2	352	32.3	32.6	23.8	20.8	26.1	43.5	33.1	74.9	100.0
VP3	860–1561	+2	233	34.1	35.9	24.5	20.3	15.1	41.3	33.7	72.5	100.0
ST antigen	5028–4459	−1	189	35.1	34.0	39.5	34.6	40.1	42.5	31.8	81.0	100.0
LT antigen	5028–4792, 4437–2632	−1	680	40.4	41.2	44.2	42.0	39.9	49.3	40.0	80.5	100.0

*IPPyV, Institut Pasteur polyomavirus; JCV, JC polyomavirus; BKV, BK polyomavirus; KIV, KI polyomavirus; WUV, WU polyomavirus; MCyV, Merkel cell polyomavirus; TSV, trichodysplasia spinulosa–associated polyomavirus; SV40, Simian virus 40; LPV, lymphotrophic polyomavirus; HPyV9, human polyomavirus 9; VP, viral protein; ST antigen; small T antigen; LT antigen, large T antigen. Pairwise sequence alignment was performed by using EMBOSS Needle Software (http://emboss.sourceforge.net/apps/release/5.0/emboss/apps/needle.html).

### Phylogenetic Analysis

**Figure 2:**
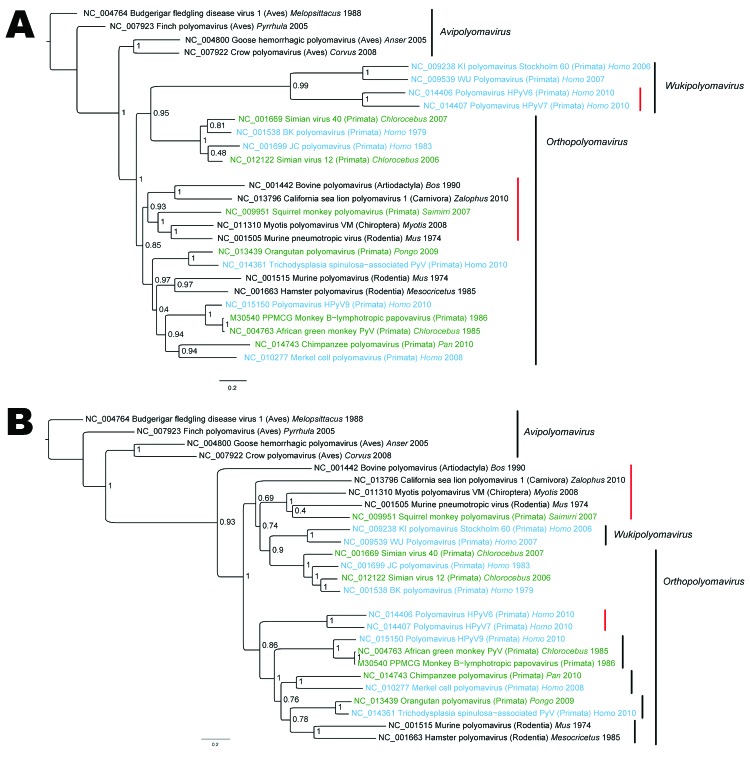
A) Viral protein 1 (VP1) and B) large T antigen (LT) nucleotide-based phylogenetic reconstructions of polyomaviruises inferred by using a Bayesian method. Taxa annotations include reference number, name of the virus, host taxonomic order (in parentheses), host genus whenever available, and reported collection date. Human viruses are indicated in blue, and monkey viruses are indicated in green. Red vertical bars highlight groups for which VP1 and LT signals are incongruent. Posterior probabilities are indicated at each node. GenBank identification numbers are indicated directly on trees for each sequence. Scale bars indicate nucleotide substitutions per site.

Reconstructions of VP1 and LT phylogenies on the basis of nucleotide sequences clustered the mammalian polyomaviruses and placed the species *Avipolyomavirus* in basal position when rooting with the oldest known *Avipolyomavirus* (Budgerigar fledging virus) (Figure 2). Despite the divergences described below, VP1 and LT of HPyV9 were closely related to those of the monkey B-lymphotropic polyomavirus. Moreover, VP1 and LT phylogenies consistently identify several monophyletic groups among mammalian polyomaviruses (Figure 2). Nevertheless, the incongruence of VP1 and LT signals induce notable differences in the topology of these 2 phylogenies. For instance, LT of bovine polyomavirus is closely related to one of the common ancestors of all other mammalian polyomaviruses, whereas its VP1 is closely related to VP1 of the sea lion polyomavirus. Incongruence between VP1 and T phylogenies has been observed for HPyV6 and HPyV7. Nomenclature described in proposals of the International Committee on Taxonomy of Viruses is shown in Figure 2, even though the species *Orthopolyomavirus* is not monophyletic and therefore should be considered cautiously.

### Comparison of VP1 from LPV and HPyV9

**Figure 3:**
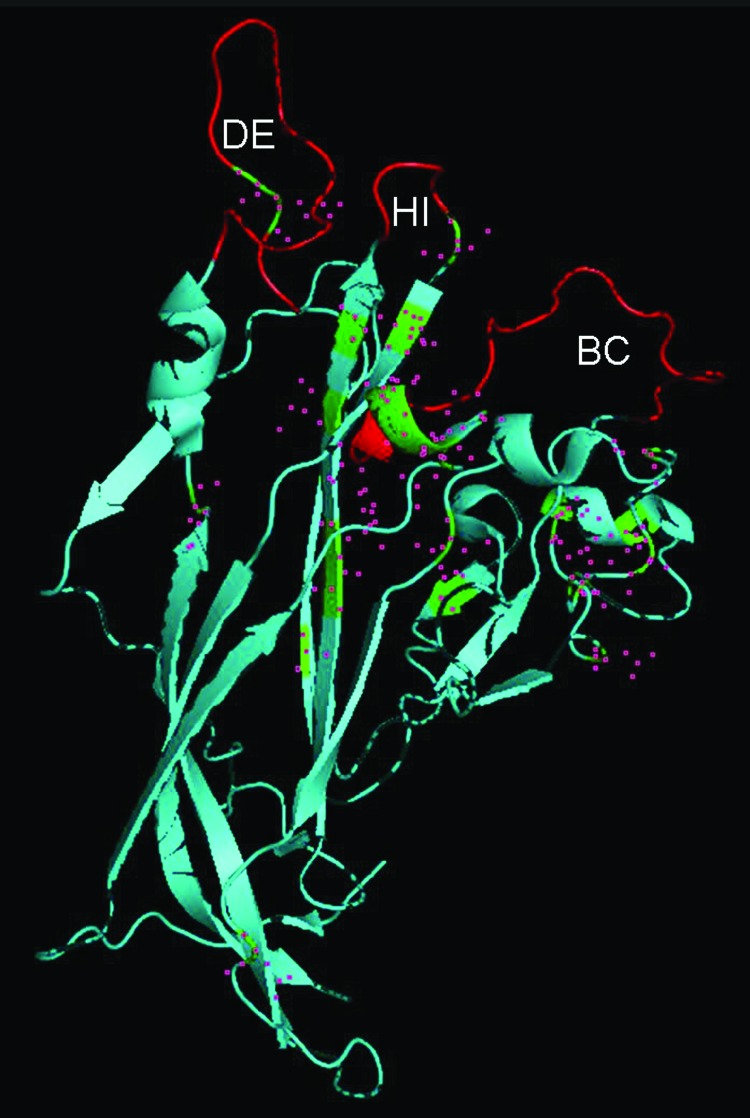
Identification of viral protein 1 (VP1) residues differing between human polyomavirus 9 (HPyV9) and lymphotropic polyomavirus (LPV). The DE, HI, and BC loops that extend outward from VP1 are indicated. The crystal structure of simian virus VP1, derived from strain 3BWQ, was used as a template. The red region in the center indicates part of a β strand, which is mostly hidden. Residues differing between HPyV9 and LPV are indicated by pink squares.

We compared the secondary structure of VP1 from LPV and HPyV9 because the external capsid protein of *Polyomaviridae* is known to interact with the cell receptor and because antibodies cross-reacting with LPV VP1 have been detected in a large proportion of humans. Overall amino acid identity was 87.1%. The VP1 monomer consists of antiparallel β-strands folded into a jelly roll β-barrel structure. Three outer loops (BC, DE, and HI) are exposed outside the pentamer core and are most likely recognized by antibodies. Using the crystal structure of SV40 VP1 (3BWQ), we mapped the amino acids that differed between the 2 proteins. Polymorphic residues are present in the 3 VP1 loops, and the BC and HI loops appear more conserved than DE loop, which thus shows the major differences (Figure 3).

### Detection of HPyV9 in Human Samples

We first confirmed by specific nested PCR the presence of HPyV9 in the skin swab specimen of the index patient in which the virus had been identified by HTS. We also identified this virus by nested PCR in a second cutaneous sample from the same index patient case obtained 20 months after the first sample. Because HPyV9 was identified in a patient who had MCC, we analyzed the skin surface of 7 other MCC patients. HPyV9 was detected in 1 patient 80 years of age. The overall prevalence in the MCC group was thus 2/8 (25%, 95% confidence interval 4.4%–64%; p = 0.05).

Because we were interested in possible interhuman transmission of HPyV9, a skin swab specimen from the wife of the first index patient was analyzed and found to be positive for this virus. We sampled 111 skin swab specimens from healthy persons or non-MCC patients who had no known contact with MCC patients and screened them with the same nested PCR. Only 1 healthy 30-year-old person had HPyV9, which demonstrated low prevalence in this control group of 1/111 (0.9%, 95% confidence interval 0.05%–5.6%; p = 0.05). Age range of the MCC population was 57–86 years (median 75 years), and because we were unable to detect HPyV9 among the 30 older controls (age range 57–96 years, median 71 years), this finding suggests that the rate of detection in MCC samples was not biased by older ages of these patients. None of the 92 respiratory and 92 stool specimens was positive for HPyV9.

## Discussion

There is much evidence indicating that healthy human skin harbors numerous viruses. This finding has been extensively reported for cutaneous human papillomavirus (HPV), which is commonly present on the superficial layers of the skin of most persons (*11*). The recent description of new human viruses belonging to the family *Polyomaviridae* suggests that some of these viruses share the cutaneous tropism of β-HPV and γ-HPV. MCPyV associated with MCC has also detected on the surface of healthy skin of most persons (*5**,**12*), and HPyV6 and HPyV7 have been identified on the surface of the skin of healthy persons (*13*). Detection of an additional human polyomavirus in cutaneous samples reinforces the perception of the skin as a complex microecosystem colonized by many viruses, and polyomaviruses represent part of this viral microbiota.

The existence in humans of a polyomavirus closely related to LPV, whose natural host is the African green monkey, has been anticipated (*14*). An African green monkey polyomavirus, also known as monkey B-lymphotropic papovavirus, or LPV, was isolated >30 years ago (*15*) from a lymphoblastoid cell line derived from the African green monkey. The existence of a virus closely related to LPV has been suspected for >30 years because of cross-reacting antibodies in humans who had no known contact with monkeys (*14**,**16*). PCR amplification of short sequences matching those of LPV has been reported, but the length and overlap of these sequences were insufficient for characterizing this virus (*13**,**17*). The phylogenetic position of HPyV9 we described makes it the sister group of LPV. Moreover, we have shown that the sequence of this virus makes it a good candidate to be the target of antibodies found in humans.

The BC and HI loops of VP1 appear conserved between HPyV9 and LPV; the DE loop shows major changes. Thus, human antibodies cross-reacting with LPV may preferentially recognize the BC and HI loops, and antibodies specific for the DE loop probably cross-react only weakly with the LPV VP1. With the availability of HPyV9-specific reagents, analysis of the specificity of antibodies found in humans will provide useful information.

HPyVs are ubiquitous viruses that infect most of a given population, remain latent, and show frequent reactivations that lead to asymptomatic shedding (*1*). The VP1 and T proteins of HPyV6, HPyV7, and several polyomaviruses from wild animals show different pathways of evolution (Figure 2). The close relationship between the HPyV6–HPyV7 group and the WUPyV–KIPyV group could result from convergence caused by common host pressure on VP1 domains (involved in virus–host interactions) or from horizontal gene transfer (recombination).

In immunocompromised persons, reactivation of HPyV often results in specific diseases such as progressive multifocal leukoencephalopathy caused by JCPyV and tubular nephropathy and hemorrhagic cystitis caused by BKPyV. Most newly identified HPyVs are also detected frequently in healthy persons. In addition, most HPyVs (KIPyV, WUPyV, HPyV6, and HPyV7) have not been clearly associated with any human diseases; however, MCPyV has been associated with MCC (*18*) and TSPyV has been associated with a rare cutaneous disorder (*2*); both occurred in immunocompromised patients.

HPyV9 was detected in skin of healthy persons who did not have any obvious immunodeficiency and thus might have asymptomatically shed HPyVs. However, among the 3 persons in whom we detected HPyV9, 2/8 had MCC in cutaneous samples, yielding an apparent higher rate of detection (2/8) in this subset of patients, compared with 1/111 in a control and heterogeneous population. This difference in prevalence rate was not biased by age within each group (0/30 had HPyV in the age-matched part of the control group). Although this unexpected result should be interpreted with caution, given the small number of MCC samples and the retrospective nature of our analysis, MCC patients might be prone to reactivation of HPyV9 shedding from their skin or HPyV9 might be involved in the pathophysiology of MCC. We are currently exploring expression of HPyV9 LT antigen in MCC and other cancer tissues. Long-term carriage of HPyV9 for >20 months observed in the index patient with MCC is consistent with this hypothesis.

Chronic shedding of HPyV from skin is similar to a well-known feature of cutaneous HPVs that replicate in keratinocytes and are likely to be transmitted environmentally or through person-to-person contact. In our study, detection of HPyV9 in skin of the wife of the index patient suggests a similar route of transmission.

It has been proposed that MCPyV and HPyV6 or HPyV7 may infect superficial cells of the epidermis and that production of virions may be, as for HPVs, linked to differentiation of the epidermis (*3*). We cannot rule out a similar scenario for HPyV9. However, because its closest relative (LPV) has been described as a lymphotropic virus, on the basis of its in vitro growth ability, the ability of HPyV9 to infect lymphoid precursors and its putative role in various lymphoproliferative disorders in humans are worth coinvestigating.

HPyV9 was detected in cutaneous samples but not in respiratory and fecal samples, and the rate of detection appeared lower than that reported for MCPyV (*19*) or HPyV6 and HPyV7 (*3*). The sampling site was chosen because of methods useful for detection of HPyVs on the skin (*3**,**12*). Furthermore, we have observed a specific pattern for MCPyV shedding because face swab specimens yielded a higher rate of viral detection than limb specimens (*5*). However, because shedding of HPyV was not as extensively studied as that of HPVs, we cannot rule out a similar pattern of excretion, which results in underestimating detection of HPyV9 on face swab specimens. The exact prevalence of HPyV9 should be investigated by using serologic and PCR assays, notably to investigate the relevance of published data suggesting that ≈30% of humans have antibodies specific for an LPV-like virus.

Because clinical manifestations associated with HPyV infections dramatically increase in immuocompromised patients, clinical manifestations caused by HPyV9, if they exist, are also more likely to occur in these patients. Furthermore, HPyV9 infection might not remain restricted to the cutaneous area in immunocompromised patients, and reactivation might lead to systemic dissemination and in some cases clinical signs. This hypothesis is supported by identification of HPyV9 in blood and urine of asymptomatic renal transplant recipients (*4*). Further investigations will be required to decipher the potential role of HPyV9 in homeostatic and pathologic processes.
